# First High-Density Linkage Map and Single Nucleotide Polymorphisms Significantly Associated With Traits of Economic Importance in Yellowtail Kingfish *Seriola lalandi*

**DOI:** 10.3389/fgene.2018.00127

**Published:** 2018-04-17

**Authors:** Nguyen H. Nguyen, Pasi M. A. Rastas, H. K. A. Premachandra, Wayne Knibb

**Affiliations:** ^1^GeneCology Research Centre, Faculty of Science, Health, Education and Engineering, University of the Sunshine Coast, Sunshine Coast, QLD, Australia; ^2^Department of Biosciences, Faculty of Science, University of Helsinki, Helsinki, Finland

**Keywords:** genotyping by sequencing, GWAS, aquaculture, gene identification, genetic improvement

## Abstract

The genetic resources available for the commercially important fish species Yellowtail kingfish (YTK) (*Seriola lalandi)* are relative sparse. To overcome this, we aimed (1) to develop a linkage map for this species, and (2) to identify markers/variants associated with economically important traits in kingfish (with an emphasis on body weight). Genetic and genomic analyses were conducted using 13,898 single nucleotide polymorphisms (SNPs) generated from a new high-throughput genotyping by sequencing platform, Diversity Arrays Technology (DArTseq^TM^) in a pedigreed population comprising 752 animals. The linkage analysis enabled to map about 4,000 markers to 24 linkage groups (LGs), with an average density of 3.4 SNPs per cM. The linkage map was integrated into a genome-wide association study (GWAS) and identified six variants/SNPs associated with body weight (*P* < 5e^-8^) when a multi-locus mixed model was used. Two out of the six significant markers were mapped to LGs 17 and 23, and collectively they explained 5.8% of the total genetic variance. It is concluded that the newly developed linkage map and the significantly associated markers with body weight provide fundamental information to characterize genetic architecture of growth-related traits in this population of YTK *S. lalandi*.

## Introduction

Despite the economic importance of Yellowtail kingfish (YTK) *Seriola lalandi* world-wide (e.g., with a current annual production of about 4,000 tons, valuing 56 million dollars in Australia), there has been very limited published information regarding genetic and genomic architecture for quantitative complex traits in this species. Recent studies generated initial genomic resources to examine genetic diversity of YTK populations (Premachandra et al., 2017a; Sepulveda and Gonzalez, 2017) or assessing predictive power of statistical models used for genomic selection (Nguyen et al., 2018), as well using transcriptome information to identify genes related to fish immunity (Jacobson et al., 2017) and those involved with skeletal deformity during early growth phase in this species (Patel et al., 2016). To date, only a low-density genetic linkage map was developed for *S. lalandi*, involving a handful of 217 microsatellites markers (Ohara et al., 2005). Potential marker candidates associated with traits of commercial importance, however, have not been reported for this species *S. lalandi*.

By contrast, genome-wide association studies (GWAS) in human and agricultural species have been successful in identifying loci contributing to genetic variations in a range of human complex diseases and quantitative traits. For instance, close to 700 variant markers were detected involved with human height and they all together explain about 15% of total variation (Wood et al., 2014). In aquaculture, the associations of DNA markers (microsatellites or SNPs) with growth and carcass traits (Gonzalez-Pena et al., 2016), disease resistance (Correa et al., 2016; Palaiokostas et al., 2016) or sexual maturity (Gutierrez et al., 2015) were reported in a number of species of commercial importance, such as salmonids and seabream. Results from these studies showed that growth or disease resistance trait is generally of polygenic nature (controlled by multiple loci, each with small effects) or they possess oligogenic architecture with a few moderate-large effects quantitative trait loci (QTL) (Vallejo et al., 2017). There are exceptions where a single locus QTL had a very strong effect on infectious pancreatic necrosis virus in Atlantic salmon (Moen et al., 2009; Houston et al., 2010). However, there is a paucity in our knowledge regarding marker effects on traits of economic importance in YTK *S. lalandi*. QTL were reported to reside on two different linkage groups (LGs) for the monogenean fluke ectoparasite (*Benedenia seriolae*) but in a different kingfish species, i.e., *Seriola quinqueradiata* (Ozaki et al., 2013).

Therefore, we conducted this study with two main goals: (i) to develop a new generation of high-density linkage map for YTK *S. lalandi*, and (2) to identify loci contributing to variation in quantitative complex traits of YTK (focusing on body weight). The study used a new high-throughput genotyping by sequencing (Diversity Arrays Technology, DArTseq^TM^) that enables to simultaneously genotype hundreds of thousands of single nucleotide polymorphisms (SNPs). The DArTseq^TM^ technology (Kilian et al., 2012) represents a combination of a DArT complexity reduction methods and next generation sequencing platforms.

Our results showed that a high-density linkage map can be constructed using the markers generated by the DArTseq^TM^ platform. A number of markers were significantly associated with growth. These markers are potentially linked with genes with known functions for growth. The findings from this study provide information for future fine mapping, gene mining, polymorphism identification of causative SNPs and their pathways and biological functions related to complex traits of economic importance in YTK.

## Materials and Methods

### Animals

The animals used in this study came from a genetic line of YTK selected for high growth at Clean Sea Tuna Ltd. in South Australia since 2010 (Whatmore et al., 2013; Knibb et al., 2016; Nguyen et al., 2016). They were progeny of 16 sires and 31 dams produced in 2010. A detailed description from breeding, nursing to harvest is given in our previous studies (Knibb et al., 2016; Nguyen et al., 2018). Briefly, breeding was conducted in tank, comprising three males and three females. After a nursing/rearing period of about 120 days in tanks, fingerlings were transferred to culture in sea cages. When the fish reached an average body weight of 3 kg, they were harvested and then anesthetized using clove oil and cold water before morphometric measurements were made and fin tissues were collected. The tissue samples were collected from caudal fin of each fish and stored in 80% ethanol for DNA extraction at a laboratory of the University of the Sunshine Coast (USC).

### Traits

Three traits of commercial importance analyzed in this study were body weight, skin fluke and morphological deformity. Body weight showed moderate to high heritability, whereas skin fluke and deformity were lowly heritable (Whatmore et al., 2013; Premachandra et al., 2017b).

Skin fluke is due to the monogenean fluke parasite *Benedenia seriolae*; this fluke inhabits the skin and fins of *Seriola* spp. and feeds on mucus and epithelia cells (Premachandra et al., 2017b). Due to the small proportion of each deformity type, this trait (deformity as defined here) included a range of measures, namely deformed snout, water belly – a condition where the belly is distended, deformed tail, deformed operculum and lower jaw (Nguyen et al., 2016).

Both deformity and fluke were recorded as binary traits depending on their presence or absence on the body of the fish at harvest (∼3 kg) and coded as 1 and zero, respectively.

The incidence of skin fluke and deformity recorded under field condition was low in this population (4.3 and 17.6%, respectively); hence genome-wide association analysis results were not tabulated in the present report. They were, however, included as **Supplementary Files S4–S7**.

### DNA Extraction, Genotyping, and Parentage Analysis

DNA isolation and amplification were performed following standard protocols in our laboratory (Premachandra et al., 2017a). A panel of eight microsatellite markers were used to genotype the experimental fish (Whatmore et al., 2013; Knibb et al., 2016). PCR products were separated by capillary electrophoresis on an AB 3500 Genetic Analyzer (Applied Biosystems). Fragment sizes were determined relative to an internal lane standard (GS-600 LIZ; Applied Biosystems) using GENEMARKER v1.95 software (SoftGenetics, State College, PA, United States) and double-checked manually. Parentage assignment was completed using COLONY software (Jones and Wang, 2010) with confidence scores of above 95%. The pedigree included 65 full-sib groups (16 dams and 31 sires), with the family size of 3–108 offspring. A total of 1,957 offspring out of 1,998 individuals were assigned to full- and half-sib families (Premachandra et al., 2017b). In this study, representatives of large size families were sent to DArT Ptd. Ltd. in Canberra, Australia for sequencing. Seven hundred and fifty-two individual fish sequenced by DArT-seq technology platform contained 35 (full and half-sib) families varying in size between 2 and 39 (average family size = 17 fish).

### Sequencing

Library preparation and DArTseq^TM^ sequencing of YTK samples is given in Nguyen et al. (2018). Briefly, four methods of complexity reduction were tested (Kilian et al., 2012) and the PstI-SphI method that corresponds to two different restriction enzymes (REs) was selected. The PstI-compatible adapter was designed to include Illumina flow-cell attachment sequence, sequencing primer sequence and “staggered,” varying length barcode region, similar to the sequence reported by Elshire et al. (2011). Reverse adapter contained flow-cell attachment region and SphI-compatible overhang sequence. Only “mixed fragments” (PstI-SphI) were effectively amplified in 30 rounds of PCR. After PCR, equimolar amounts of amplification products from each sample of the 96-well microtiter plate were bulked and applied to c-Bot (Illumina) bridge PCR followed by sequencing on Illumina Hiseq2500. The sequencing (single read) was run for 77 cycles.

Sequences generated from each lane were processed using proprietary DArT analytical pipelines. Approximately 2,500,000 sequences per barcode/sample were identified and used in marker calling. For SNP calling, all tags from all libraries included in theDArTsoft14 analysis are clustered using DArT PL’s C++ algorithm at the threshold distance of 3, followed by parsing of the clusters into separate SNP loci using a range of technical parameters, especially the balance of read counts for the allelic pairs. Additional selection criteria were added to the algorithm based on analysis of approximately 1,000 controlled cross populations and Mendelian testing of alleles distribution. In addition, multiple samples were processed from DNA to allelic calls as technical replicates and scoring consistency was used as the main selection criteria for high quality/low error rate markers. Calling quality was assured by high average read depth per locus (Average across all markers was over 30 reads/locus). The marker call rate was 98% and the sample call rate was 92%. In this study, about 30% of the samples (*n* = 226) were processed more than once from DNA to marker call and calling consistency was evaluated and reported for each SNP (ranging from 90 to 100%, average 98%). The raw sequence data has the accession number SRP130211.

### Linkage Analysis

The linkage maps were constructed using Lep-Map3 software (Rastas et al., 2016).

First, the genotypes were converted to genotype likelihoods (posteriors) using linkage2post.awk script provided with Lep-MAP3. Then the missing parental genotypes were imputed using ‘ParentCall2’ module. The half-sib families were taken into account by iterating ‘ParentCall2’ module three times with option outputParentPosteriors = 1 following the joining the parental genotype likelihoods for the identical parents. For the final dataset, the parental genotypes were called without outputParentPosteriors option.

The resulting data was filtered by comparing the offspring genotype distribution and the expected Mendelian proportions using the ‘Filtering2’ module (parameter ‘dataTolerance’ was 0.001) to remove markers that exhibited significant segregation distortion (χ^2^ test, df = 1–2 and *P* < 0.001, i.e., one out of 1,000 markers were removed by chance, separately for each family).

The ‘SeparateChromosomes2’ module was then applied to cluster markers into LGs, with the LOD threshold of 30 (lod Limit parameter) and recombination rate 0.05 (theta parameter) forming 24 LGs (the expected karyotype of YTK, 2*n* = 48). Subsequently, the module ‘JoinSingles2All’ was performed to assign additional single SNPs to existing LGs using LOD threshold 20. Finally, the markers were ordered using ‘OrderMarkers2’ module with the default parameters. To verify the correctness of the marker order within each LG, the module ‘LMPlot’ was used to produce Lep-MAP plots for each chromosome. Sex-specific map distances were calculated in centiMorgans (cM) using the Haldane mapping function. Sex-differences in recombination rate between male and female in YTK are reported in Section “Data.”

### Genome-Wide Association Study (GWAS)

Genome-wide association study analyses were conducted using four different statistical methods: (1) genotype association/correlation test (GAT), (2) correlation test using principal component analysis (PCA) to correct for stratifications (e.g., population structure), (3) numeric regression with the PCA corrections, and (4) mixed model methods.

The GAT assumes that all samples in the analysis are unrelated and selected from a uniform, random-mating population. This was not the case in this study. Hence, the PCA was used to stratify samples based on genomic similarity, as well to assess cryptic relatedness in the population.

Both single and multiple locus models (Kang et al., 2010) were used. The mixed model is more powerful than PCA-based correction since it can account for pairwise allele sharing among all study samples. In this study, the mixed model GWAS included the fixed effect of stock origin (wild and captive) and a kinship matrix as a random factor. The identity-by-state (IBS) kinship matrix was computed from the genotypic data (13,898 markers). The multiple locus model also used a forward and backward stepwise approach to select markers as fixed effect covariates in the model. In a matrix notation, the mixed model is written as:

(1) $$\begin{array}{c} y\;=\;Xb+Ga+e \end{array}$$

where ***y*** is the vector of observations for traits studied (weight, deformity, and skin fluke), ***X*** is the incidence matrix related to the fixed effects. ***b*** is the vector of all possible systematic fixed effects, including stock origin (wild or domestication). Vector ***a*** is the random animal additive genetic effects ∼ (0, **A**$\sigma_{a}^{2}$) where ***G*** is the genomic relationship matrix calculated from the SNP markers, and ***e*** is the vector of residual effects ∼ (0, **I**$\sigma_{a}^{2}$).

**FIGURE 1:**
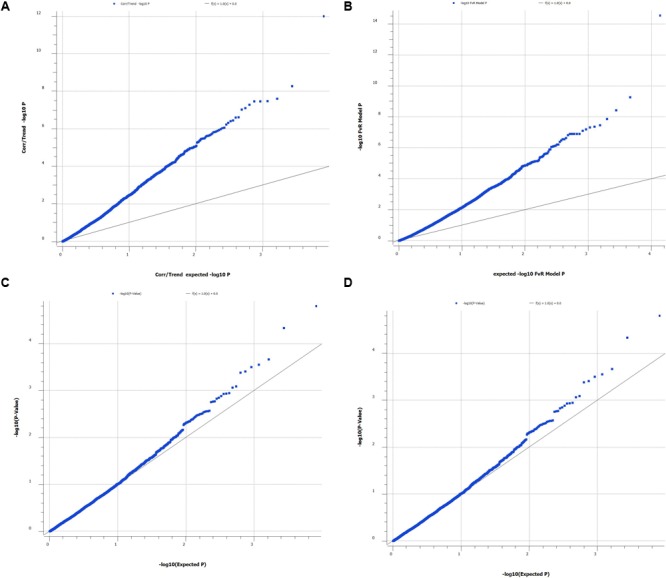
Quantile-quantile plot of P-values from single SNP genome wide association study (**A**, correlation/trend test and **B**, numeric regression with corrections for PCAs (**C**, single locus mixed model and **D**, multiple locus mixed model). Lambda values for **(A–D)** are 2.23, 2.06, 1.01, and 0.961, respectively.

We also used Q–Q plot to assess possible effects of the population structure and cryptic relatedness that may cause biases in the results (**Figure 1**). In addition, we calculated multiplicative inflation factor [λ = median(X_i_^2^)/0.456] where X_i_^2^ is the distribution of the chi-square statistic over a set of markers or over the actual association tests. The lambda value λ > 1 indicates population substructure or genotyping errors (SNPs with λ > 1 was omitted).

Multiple testing corrections used both Bonferroni and false discovery rate (FDR) methods to minimize type I errors (i.e., SNPs declared were significant but they were not present). In this study, about 13,898 markers were analyzed, the corrected significant level was set at 0.3 × 10^-5^. However, the Bonferroni test is known to be associated with high probability of type II errors (fail to detect significant SNPs that are present). Hence, the FDR was also used to control the proportions of false positives. The genome-wide significance thresholds (1%) based on 1,000 permuted datasets were also used to screen significantly associated SNPs in SVS Suite (Golden Helix).

## Results

### Data

The mean body weight of this YTK population was around 3 kg (*SD* = 0.35). The quantitative genetic variation analysis in ASReml showed that body weight was moderately heritable (*h*^2^ = 0.42).

In total, there were 13,898 SNP markers. The polymorphism information content (PIC) value for the SNPs was 0.16 under additive genetic model, whereas it was substantially higher, 0.46 under codominant model. The average proportion of missing genotype (SNPs) in the raw data (before QC) was only 14.8%. The average heterozygosity proportion of SNPs ranged from 10 to 30%, with a mean value of 10%. The frequency of minor allele was 0.29.

After data filtering to remove significant distortion markers, 3,998 SNPs remained and they were used to construct linkage maps. The PIC value of the SNP allele was 0.18. The average PIC of the reference and SNP alleles was 0.10. The average proportion of homozygous and heterozygous SNPs was 0.14 and 0.08, respectively.

### Linkage Maps

#### Female, Male, and Sex-Averaged Maps

**FIGURE 2:**
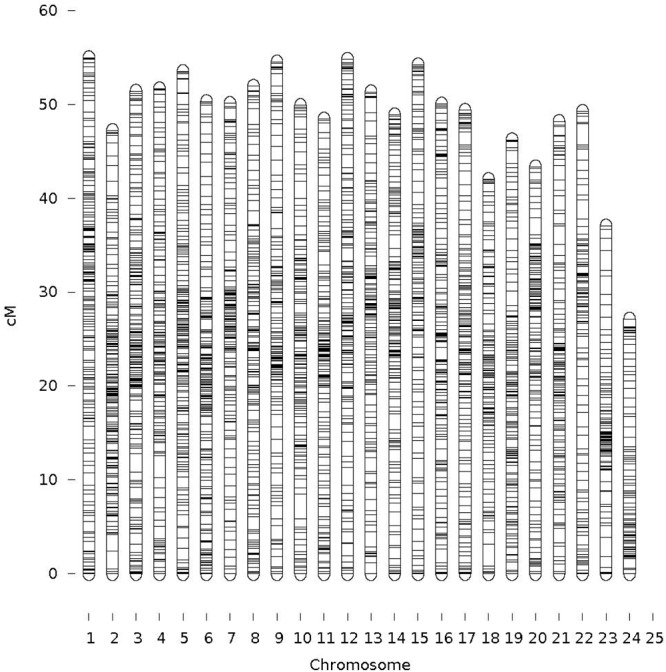
Sex averaged linkage map (specific-linkage maps included in **Supplementary File S3**).

A total of 3,998 SNPs were mapped to 24 LGs, which correspond to the YTK karyotype (Chai et al., 2009). The total map length was 1,166 cM (**Table 1**). The remaining SNPs markers (9,998) were not placed on the 24 LGs during mapping due not having enough informative families and individuals (both before and/or after filtering). The male and female genetic maps were comparable with total lengths of 1,136 cM and 1,197 cM, respectively. The range of SNPs density per chromosome was from 101 to 205 (mean = 167 and SD = 26.8). The LG lengths varied between 27.12 and 54.83 cM (mean = 48.59 and SD = 6.09). The correlation between the number of SNPs (proxy for genome length) and corresponding chromosome map length was 0.75. The sex-average marker map is presented in **Figure 2** and sex-specific marker map for female and male YTK in **Supplementary File S3**. The actual marker positions for each sex or both female and male combined are given in **Supplementary File S1**.

**Table 1:** Summary statistics of the sex-averaged linkage map of yellowtail kingfish *Seriola lalandi.*

LG	Number of markers	Size (cM)			Average distance between markers (cM)		
	Average	M	F	Average	M	F	Average
1	203	53.65	56.35	55.00	0.26	0.28	0.271
2	197	47.12	47.36	47.24	0.24	0.24	0.240
3	200	48.44	54.41	51.43	0.24	0.27	0.257
4	182	50.45	52.89	51.67	0.28	0.29	0.284
5	205	46.82	60.23	53.53	0.23	0.29	0.261
6	190	47.21	53.43	50.32	0.25	0.28	0.265
7	177	50.40	49.85	50.13	0.28	0.28	0.283
8	180	52.01	51.89	51.95	0.29	0.29	0.289
9	175	52.79	56.27	54.53	0.30	0.32	0.312
10	170	46.65	53.17	49.91	0.27	0.31	0.294
11	168	39.53	57.36	48.45	0.24	0.34	0.288
12	175	52.4	57.25	54.83	0.30	0.33	0.313
13	164	49.72	53.00	51.36	0.30	0.32	0.313
14	185	49.42	48.4	48.91	0.27	0.26	0.264
15	156	45.48	62.97	54.23	0.29	0.40	0.348
16	165	50.12	49.98	50.05	0.30	0.30	0.303
17	167	43.73	55.04	49.39	0.26	0.33	0.296
18	153	37.88	46.15	42.02	0.25	0.30	0.275
19	161	41.22	51.26	46.24	0.26	0.32	0.287
20	147	49.31	37.40	43.36	0.34	0.25	0.295
21	147	46.98	49.43	48.21	0.32	0.34	0.328
22	121	53.2	45.33	49.27	0.44	0.37	0.407
23	109	35.33	38.76	37.05	0.32	0.36	0.340
24	101	45.78	8.46	27.12	0.45	0.08	0.269
Total	3998	1136	1197	1166	6.99	7.17	7.08
Average	167	47.32	49.86	48.59	0.291	0.299	0.30

*LG, linkage group; M, male; F, female. The number of markers come from the same maps and thus had the same numbers for female and male.*

#### Recombination

The female to male recombination ratio was 1.03:1. The highest recombination ratio between sexes was observed on LG15 with the female to male ratio of 1.38:1, and the lowest female to male recombination ratio was on LG24 (0.18:1). Chromosome regions in which recombination occurred differed between female and male, although the differences depended on LGs. Overall, the high recombination rates were observed near the telemetric regions on the female map, such as LG1, 2 and 15, 19 and 21. For male, the high recombination rates were near the centrometric regions on LG2-4 and 6-24.

#### Segregation Distortion

The segregation distortion was based on markers and the analysis showed that there was significant segregation (*P* < 0.001) in some individual families (**Supplementary File S2**). The distorted loci were filtered before the linkage map was constructed. At the significant probability of 0.0001, there were, however, only 44 and 22 loci showing segregation distortion on the female and male maps, respectively. In both female and male maps, the LG2 represented the most significant segregation distortions (3.5 and 2.2%, respectively), whereas 14 out of 21 LG didn’t have the distorted markers. For the sex-averaged linkage map, the LG2 also exhibited the most significant segregation distortions (1.4%). The lowest segregation distortion was observed on LG13 (0.06%). The distorted markers tended to be clustered or unevenly distributed in LGs.

### GWAS for Body Weight

**FIGURE 3:**
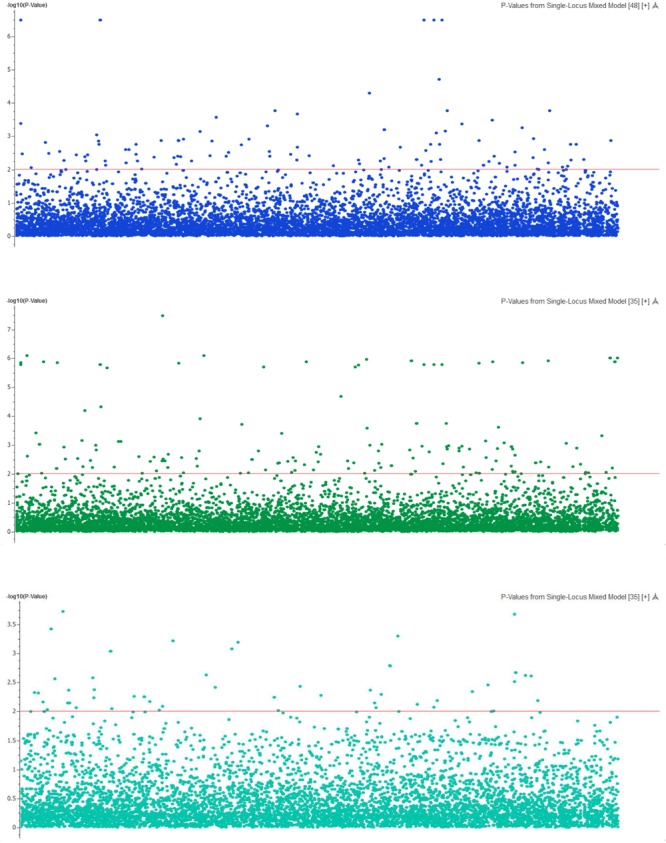
The Manhattan plot showing the –log_10_ (*P*-values) of SNPs on body weight (top), deformity (middle) and skin fluke (bottom) using single locus mixed model.

Genome-wide association analysis was conducted using the multi-loci mixed model. Significance threshold was set at the *P-*value less than 5e^-8^ or -log*P*-value > 5. At this high level of stringency, six SNPs were significantly associated with body weight (**Figure 3**), in which two loci were mapped to LGs 17 (CloneID: 13793879|F| 0-12:A>G-12:A>G) and 23 (13791643|F| 0-7:G>A-7:G>A), respectively. Interestingly these SNPs were also detected from three other statistical methods: GAT, GAT with a correction for the first three components from principal coordinate analysis (PCA) and PCA-based numeric regression (**Table 2**).

**Table 2:** Number of SNPs (N) and their false discovery rates (FDRs) (%) for body weight (*P* < 5e^-8^ or -log_10_*P* > 5).

Trait	Correlation		With PCA corrections		Regression with PCA corrections		Single locus mixed model		Multiple locus mixed model	
	*N*	FDR	*N*	FDR	*N*	FDR	*N*	FDR	*N*	FDR
Weight	80	0.5 × 10^-4^	68	0.5 × 10^-2^	63	0.5 × 10^-2^	6	0.009	6	0.009

**FIGURE 4:**
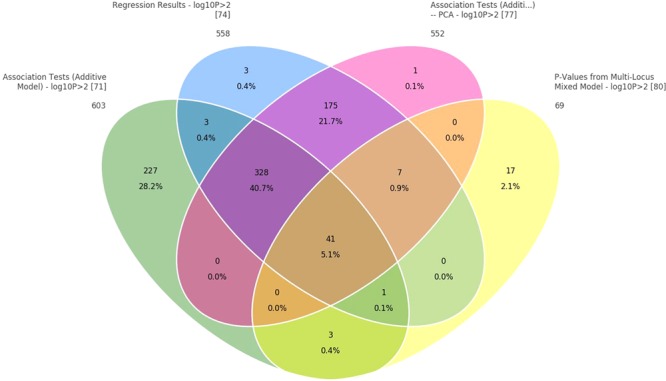
Venn diagram to show common SNPs among the four methods: (1) correlation/trend test, (2) correlation/trend test with corrections for PCAs, (3) numeric regression with corrections for PCAs, and (4) mixed model to correct for cryptic relationship.

In addition, when the data were analyzed with GAT and PCA-based correction methods, a larger number of significant SNPs were found to be associated with body weight (24, 19, and 14, respectively) relative to those obtained from the mixed model (**Table 3**). Those associated with body weight were mapped to 10 different LGs (**Supplementary File S3**). Two common LGs are LG10 and LG18. The proportion of common SNPs identified from the four statistical models used is given in a Venn diagram, **Figure 4**.

**Table 3:** Highly significant markers associated with traits studied, using mixed model methodology (*P* < 5e^-8^ or -log_10_*P* > 5).

Trait	Marker	LG	Position (bp)	*P*-value	MAF	Allelic effect
**Weight**						
	13793879|F| 0-12:A>G-12:A>G	17	29.2	3.27E-07	0.002	4.1
	13791643|F| 0-7:G>A-7:G>A	23	13.7	3.27E-07	0.002	4.12
	13784986|F| 0-35:A>G-35:A>G	NA	NA	3.27E-07	0.002	4.12
	13785509|F| 0-23:G>C-23:G>C	NA	NA	3.27E-07	0.002	4.12
	13801051|F| 0-36:T>A-36:T>A	NA	NA	3.27E-07	0.499	4.12
	13799009|F| 0-66:C>G-66:C>G	NA	NA	1.98E-05	0.203	4.12

*NA, not available; MAF, minor allele frequency.*

The mixed model analysis of a subset comprising 4,320 markers after a very high stringent quality control also detected three significant SNPs with –log_10_*P*-value > 4 (*P*-value = 1.5 × 10^-5^). Three other SNPs associated with body weight at the -log_10_*P*-value > 3 were also mapped to LG5 (20269726|F| 0-65:A>C-65:A>C), LG15 (13795114|F| 0-43:C>T-43:C>T) and LG18 (13791421|F| 0-24:C>A-24:C>A), respectively. Twenty-nine new markers were also detected at -log_10_*P*-value > 5 when GAT and PCA-based methods were analyzed (unpresented results) in which 10 SNPs were mapped to LGs 4, 10, 12, 14, 15, 18, and 20.

GWAS analysis results for deformity and skin fluke were included in **Supplementary Files S5** and **S6**, respectively.

## Discussion

The present study reports, for the first time in YTK: (i) a high-density SNP-based linkage map, and (ii) a set of markers significantly associated with traits of economic importance (specifically body weight).

### Genetic Linkage Maps

We developed the first dense SNP-based linkage map for YTK, containing 4,000 markers mapped to 24 LGs with 1,166 cM span (average marker interval of 0.30 cM). The high-density genetic map for YTK was constructed from a pedigreed population comprising 37 families and 666 individuals, whereas almost all earlier studies in farmed aquaculture species used crosses from diverse genetic origins. The previous linkage map reported for YTK was based on 217 microsatellites markers (Ohara et al., 2005) and generally it lacks the necessary resolution for successful implementation of marker-assisted selection (MAS) in this species. Furthermore, the marker map that was developed from crossing populations also limits possibilities of commercial applications due to differences in the genetic background and the inconsistent association between markers and QTL across populations. As observed in fish species, the pattern of recombination across the genome was notably different between the sexes, with female recombination rates being higher across many regions of the genome (Tsai et al., 2016). However, in the present population of YTK, the difference was small.

In developing the linkage map, we removed the distorted markers with *p* < 0.001 to prevent any possible bias that may result from genotyping and parental genotypes errors. Segregation distortion is reported due to accumulation of recessive deleterious mutations, genetic load, duplicated genes, transposable elements, and unusual meiotic segregation distortion. Gametic incompatibility or reduced hybrid viability also causes uneven transmission of alternate alleles, which is frequently caused by disrupted genetic interactions among loci of parental lineages, resulting in the non-random elimination of particular allelic combinations. The low segregation distortion rate in this study (averaging 2.7% across the two sexes) may have been due to the absence of the aforementioned factors in this study. In addition, the uneven distribution of the distorted markers among LGs also suggest that marker distortion was not caused by technical limitations or other typing errors. The segregation distortion rate was not reported in recent studies for marine finfish that used similar genotyping by sequencing technology. However, the segregation distortion rate was moderate to high, ranging from 17 to 66% in oyster species (Wang et al., 2016). More importantly, the linkage map constructed was integrated in our GWAS analysis for complex traits measured in this study.

### Genome-Wide Association Study (GWAS)

Genome-wide association study analysis of 777 genotyped kingfish in which 752 individuals had phenotypic records indicated that there are markers associated with body weight. The number of markers, however, depend on statistical methods used. Q–Q plots together with lambda (λ) values provided evidence that GAT and PCA-based correction methods deviated from the assumption for a standard GWAS statistical analysis that the samples are unrelated and selected from a uniform, random-mating population. As a consequence, the number of significant markers associated with body weight obtained from the GAT and PCA-based correction methods was overestimated in this study. On the other hand, the mixed model was observed as goodness of fit, the observed values lied in the expected line and the lambda value closed to one. The results obtained from the mixed model are thus expected with minimum bias because this method accounted for genetic relationship in the pedigree, as well possible systematic fixed effects.

At a very high level of stringency (*P* < 5e^-8^ or -log_10_*P* > 5), six markers were significantly associated with growth. Collectively they explained 32% of total genetic variance. GWAS studies in other aquaculture species reported significant QTLs involved with growth; however, they generally explained only a limited proportion of genetic variance, such as from 1.0 to 1.5% in rainbow trout (Gonzalez-Pena et al., 2016) and 0.02–0.08% for head size (length and width) in Channel catfish (Geng et al., 2016).

In addition, we found that 28 markers were significantly associated with deformity (**Supplementary File S5**). To date, there is no comparative literature for deformity in any aquatic animal species. Reports in terrestrial livestock species indicated that QTL regions on chromosome 26 are associated with bone related diseases (Rafati et al., 2016; Sevane et al., 2016). However, no markers were detected to be associated with skin fluke (**Supplementary File S6**). It was partially due to a small proportion of diseased fish in this study (only 4%) or by recombination during the course of line development or lack of sufficient LD between markers and the disease genes. By applying a similar genotyping by sequencing technology, Palaiokostas et al. (2016) also didn’t find any significant markers associated with Pasteurellosis in gilthead seabream. On the other hand, Ozaki et al. (2013) reported two major QTLs on the LGs 1 and 2 that explained 33–36% of total phenotypic variance for Monogenean parasite (*Benedenia seriolae*) in a different kingfish species (*Seriola quinqueradiata*). Significant QTLs were detected in other aquaculture species for a range of diseases from sea lice (Correa et al., 2016) to bacterial cold water disease (Campbell et al., 2014; Palti et al., 2015; Vallejo et al., 2017). Generally, the association of QTLs with complex traits is varied with populations and families within the same population. It is well known that statistical power of GWAS is affected by many factors, including: complexity of the genetic architecture of the phenotype, frequency and effect size of the disease allele, accuracy of phenotypic measurements and homogeneity of the phenotype, and LD relationships between causal variants and genotyped SNPs. Thus, there is a need to increase sample size to increase statistical power to detect significant associations of the markers and skin fluke in a future GWAS study for this population.

### Biological Functions of Significant Markers

In addition to the identification of multiple SNPs significantly associated with variations in growth and deformity, in the present study we found that these SNPs are located in (near) genes with known growth and immune functions, as well as in genes encoding for proteins involved in signal transduction, cytoskeleton, membrane channels and ion transport (**Supplementary Files S4, S5, S7**). Genes that are well characterized and potentially related to deformity included Interleukin-1 receptor-associated kinase 4 isoform X1 (ILK4), potassium voltage-gated channel subfamily H member 5 (KCNH2) or ribonucleases P MRP subunit POP1 (POP1). The significant SNPs that harbors genes related to growth included the e3 ubiquitin-protein ligase herc1 (HERC1), muscle-related coiled-coil (MURC) and centrosomal protein of 170 kDa isoform x1, cep170).

This suggests that growth and deformity in YTK are involved with complex processes controlled by multiple genes and gene families. Understanding genetic architecture of quantitative complex traits in conjunction with conventional selective breeding approach can accelerate genetic gain especially for characters that are difficult or expensive to measure (e.g., disease rather than growth) in YTK. Due to characteristics of the RAD-sequencing technologies, genes commonly observed in relation to growth in human (Marouli et al., 2017) or farmed animals (Bolormaa et al., 2016), such as *GHR, HMGA2, SMAD2, STC2, IGF1, LCORL, NCGPA* were not captured in this study. To date, a reference genome assembly is not available for YTK; hence, further studies are needed to gain better understanding of biological functions of the significant SNPs identified here. It is also important to validate the associated SNPs in other populations of YTK. Whole genome sequencing is expected to have greater power to detect variants that contribute to variation in quantitative complex traits as included in our analysis here.

## Conclusion

A high-density SNP-based linkage map was constructed for the first time in YTK *S. lalandi*. A total of about 4,000 markers were mapped to 24 different LGs which correspond to the karyotype of this species. Six SNPs were significantly associated with the quantitative complex trait studied (i.e., body weight). These markers each explained from 2.6 to 4.9% of total genetic variance in the model for growth. The mixed model should be used to account for cryptic relatedness in the population and to minimize false positives. Functional characterization of the significant markers deserves further study to elucidate their biological functions underlying growth and complex traits in kingfish.

## Ethics Statement

All the methods and experimental protocols of this study were performed in accordance with guidelines and regulations approved by the animal ethics committee of the University of the Sunshine Coast, Australia (approval number AN/A/10/51).

## Author Contributions

NN conceived and designed the experiments, analyzed the data, ran functional analysis, and prepared the manuscript. HP conducted the microsatellite markers genotyping and constructed the pedigree. PR ran linkage map analysis. WK oversaw the study, developed and managed the pedigree and the breeding program. NN, HP, and WK participated in data and tissue sample collection. All authors read and approved the manuscript.

## Conflict of Interest Statement

The authors declare that the research was conducted in the absence of any commercial or financial relationships that could be construed as a potential conflict of interest.

## Supplementary Material

FILE S1 Sex-specific maker map for female and male yellowtail kingfish.

FILE S2 The actual marker positions for each sex or both female and male combined.

FILE S3 Loci were significantly (*P* < 0.001) associated with body weight and mapped to linkage groups.

FILE S4 Gene ontology distribution by level for body weight.

FILE S5 Genome-wide association analysis and gene ontology distribution by level for deformity.

FILE S6 Genome-wide association analysis for skin fluke.

FILE S7 A full list of significant SNPs linked with candidate genes with known functions.
