# Supplementary material for: First High-Density Linkage Map and Single Nucleotide Polymorphisms Significantly Associated With Traits of Economic Importance in Yellowtail Kingfish Seriola lalandi
Source: Front Genet. 2018 Apr 17;9:127. doi: 10.3389/fgene.2018.00127 (PMC5914296; doi:10.3389/fgene.2018.00127)
Supplement: FILE S1 — Sex-specific maker map for female and male yellowtail kingfish. [file Data_Sheet_1.zip › Data Sheet 1/Supplementary Files S1-7/Supplementary file S4. Gene Ontology and biological functions of significant SNPs for body weight.docx]

**Supplementary file S4. Biological functions of significant SNPs associated with body weight**

**General analysis**: First, DNA sequences of SNPs significantly associated with growth were directly compared with the nucleotide collection in NCBI using BLASTn to infer their biological functions. Second, these sequences were aligned to a reference transcriptome of yellowtail kingfish (YTK). The reference transcriptome of YTK was assembled from three different tissues: muscle, ovary and liver (University of the Sunshine Coast, unpublished). The sequences that had complete (100%) match with the transcriptome (<1E-10) was then selected for functional annotation. Functional analysis was conducted in Blast2go through three main steps: Blast, InterproScan and mapping. Default settings of Blast2go were used.

**Results**: Initial blast analysis identified 37 DNA sequences of SNPs significantly associated with growth that had complete match with the YTK transcriptome. Subsequent analysis of these sequences using blast2GO obtained 12 sequences with known characterized functions. For growth, the annotated genes were related to three main processes: 1) biological (developmental, metabolic and single/ multicellular organisational processes), 2) molecular (catalytic and protein-binding activity), and 3) cellular (membrane channel and ion transports) (Figure S4). Among the annotated genes, three SNPs found in (near) genes with known growth functions included: e3 ubiquitin-protein ligase herc1 (HERC1), muscle-related coiled-coil (MURC), and centrosomal protein of 170 kda isoform x1 (CEP170)(Table S4).

**Discussion**: The significant SNPs that harbours genes related to growth included the e3 ubiquitin-protein ligase herc1 (HERC1), muscle-related coiled-coil (MURC). The HERC1 and its conserved protein domain family (WD40) have variety of functions including adaptor/regulatory modules in signal transduction, pre-mRNA processing and cytoskeleton assembly; typically contains a GH and WD40. Exome (via NGS) and Sanger sequencing reported that biallelic sequence variants in a novel gene (HERC1) might be related to overgrowth in human (Ortega‐Recalde et al., 2015). In addition, muscle-related coiled-coil (MURC) is involved in the regulation of skeletal myogenesis, including the promotion of myogenic differentiation and muscle regeneration (Tagawa et al., 2008). There are also other genes or their conserved protein domain family related to cell proliferation (centrosomal protein of 170 kda isoform x1, cep170).

| 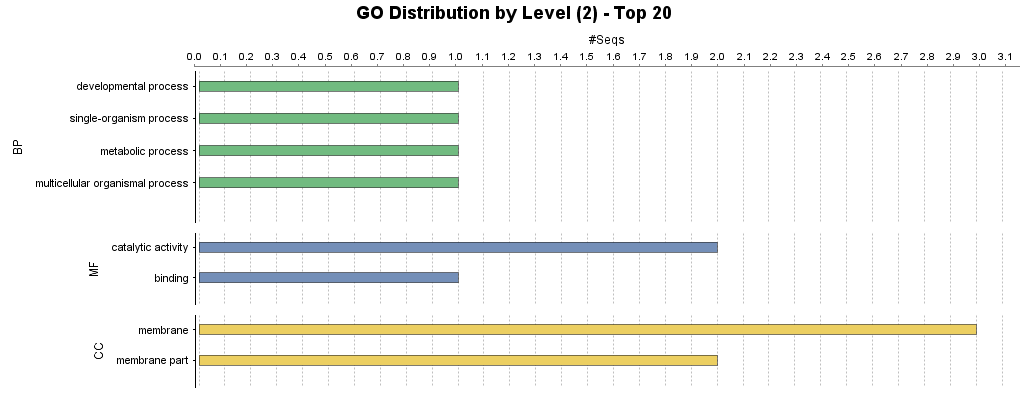 |
| --- |
| Figure S4: Gene Ontology distribution by level for body weight |

Table S4: List of significant SNPs linked with candidate genes

| Traits | SNPs | Position | Linkage group | Gene code | Gene name |
| --- | --- | --- | --- | --- | --- |
| Weight | SNP4 | 23:G>C | NA | CEP170 | Centrosomal protein of 170 kda isoform x1 |
|  | SNP19 | 15:C>T | 1 | HERC1 | Probable e3 ubiquitin-protein ligase herc1 |
|  | SNP59 | 28:G>A | NA | MURC | Muscle-related coiled-coil |

NA = Not available

**References**

Ortega‐Recalde, O., Beltrán, O., Gálvez, J., Palma‐Montero, A., Restrepo, C., Mateus, H., et al. (2015). Biallelic HERC1 mutations in a syndromic form of overgrowth and intellectual disability. *Clinical genetics* 88(4).

Tagawa, M., Ueyama, T., Ogata, T., Takehara, N., Nakajima, N., Isodono, K., et al. (2008). MURC, a muscle-restricted coiled-coil protein, is involved in the regulation of skeletal myogenesis. *American Journal of Physiology-Cell Physiology* 295(2)**,** C490-C498.
