# Supplementary material for: First High-Density Linkage Map and Single Nucleotide Polymorphisms Significantly Associated With Traits of Economic Importance in Yellowtail Kingfish Seriola lalandi
Source: Front Genet. 2018 Apr 17;9:127. doi: 10.3389/fgene.2018.00127 (PMC5914296; doi:10.3389/fgene.2018.00127)
Supplement: FILE S1 — Sex-specific maker map for female and male yellowtail kingfish. [file Data_Sheet_1.zip › Data Sheet 1/Supplementary Files S1-7/Supplementary file S5. GWAS analysis for deformity.docx]

**Supplementary file S5. GWAS analysis results for deformity**

With the mixed model analysis, 28 SNPs were found significantly associated with deformity in kingfish (P-value = 0.5×10^-2^ to 1.1×10^6^) and FDR = (0.0001 to 0.0021) (Table S5.1 and Figure S5.1), in which 8 markers were mapped to LG1, 9, 15, 17, 19 and 23 (Table S5.2). These eight markers each explained about 3.7 to 4.8% of total variance, accumulatively contributing to 26.8% of the total variation. A larger number of significant markers were detected from GAT and PCA-based correction methods. The proportion of markers that were in common with those obtained from the mixed model methodology when the –log10P-value greater than 2 and 5 was 57% and 23%, respectively.

Table S5.1: Number of SNPs (N) and their false discovery rates FDR (%) for deformity (P < 5e^-8^ or –log10P > 5)

| Trait | Correlation | | With PCA corrections | | Regression with PCA corrections | | Single locus mixed model | | Multiple locus mixed model | |
| --- | --- | --- | --- | --- | --- | --- | --- | --- | --- | --- |
|  | N | FDR | N | FDR | N | FDR | N | FDR | N | FDR |
|  |  |  |  |  |  |  |  |  |  |  |
| Deformity | 35 | 0.5×10^-3^ | 35 | 0.5×10^-3^ | 2 | 0.08 | 28 | 0.5×10^-2^ | 28 | 0.5×10^-2^ |

| 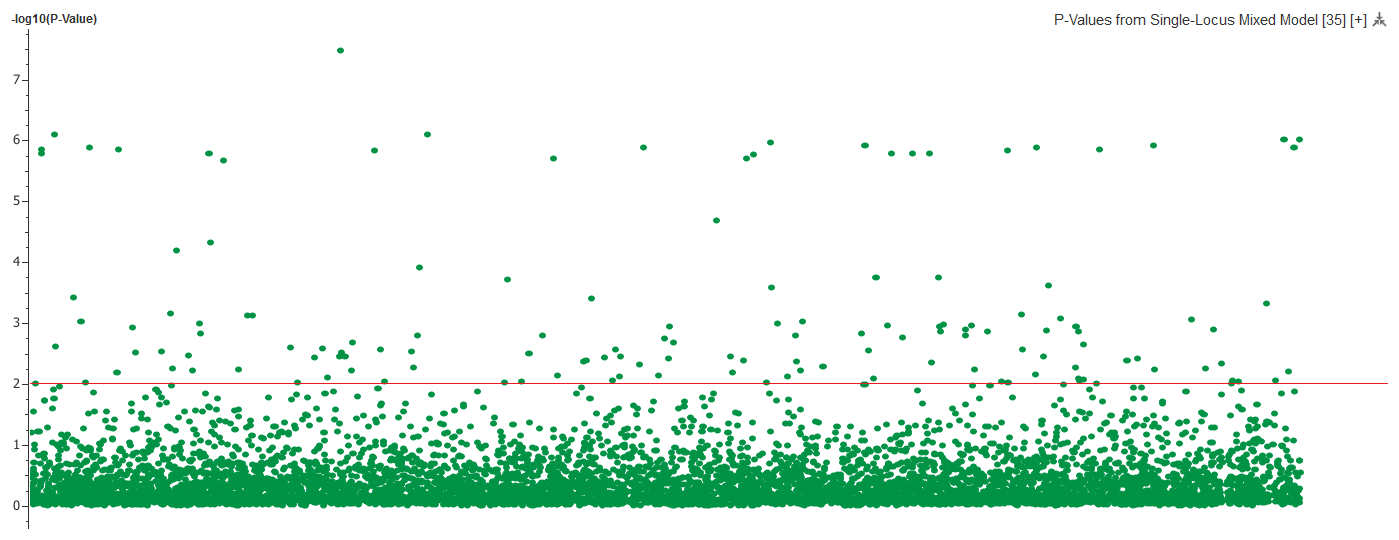 |
| --- |
| Figure S5.1: The Manhattan plot showing the –log10 (P-values) of SNPs on deformity (middle) using single locus mixed model |

Table S5.2: Highly significant markers associated with traits studied, using mixed model methodology (P < 5e^-8^ or –log10P>5)

| Trait | Marker | LG | Position (bp) | P-value | MAF | Allelic effect |
| --- | --- | --- | --- | --- | --- | --- |
| Deformity |  |  |  |  |  |  |
|  | 13796901\|F\|0-29:G>A-29:G>A | 1 | 34.9 | 3.42E-08 | 0.035 | 4.8 |
|  | 13791008\|F\|0-35:A>G-35:A>G | 9 | 23.9 | 1.10E-06 | 0.499 | 3.8 |
|  | 13801038\|F\|0-33:T>A-33:T>A | 15 | 49.5 | 1.22E-06 | 0.002 | 3.7 |
|  | 13792008\|F\|0-53:C>T-53:C>T | 19 | 34.4 | 1.48E-06 | 0.002 | 3.6 |
|  | 13793879\|F\|0-12:A>G-12:A>G | 17 | 29.2 | 1.69E-06 | 0.002 | 3.6 |
|  | 13791643\|F\|0-7:G>A-7:G>A | 23 | 13.7 | 1.69E-06 | 0.002 | 3.6 |
|  | 13800348\|F\|0-66:T>C-66:T>C | 17 | 49.4 | 2.01E-06 | 0.002 | 3.5 |
|  | 13796901\|F\|0-29:G>A-29:G>A | 1 | 34.9 | 3.42E-08 | 0.036 | 4.8 |

NA = Not available, MAF = Minor Allele Frequency

DNA sequences of the significant SNPs associated with deformity were aligned with the YTK reference transcriptome (University of the Sunshine Coast, unpublished). The reference transcripts that completely matched (i.e. 64 nt overlapped, no gaps and mismatches) with the sequences of SNPs were 32 and they were then annotated using BLASTn 2.7.1+ against the NCBI databases, in which seven sequences were characterized with known immune functions. Gene Ontology (GO) distribution of the annotated sequences is given in Figure S5.2.

| 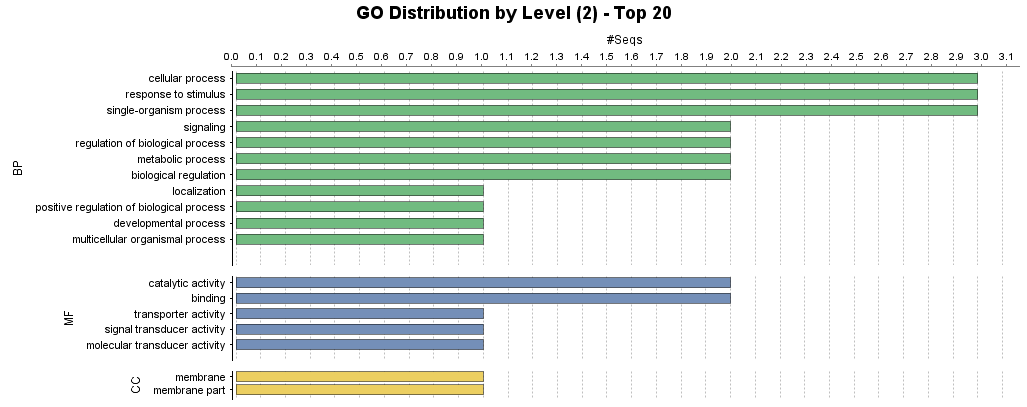 |
| --- |
| Figure S5.2: Gene Ontology distribution by level for deformity |

Genes that are well characterized and potentially related to deformity included Interleukin-1 receptor-associated kinase 4 isoform X1 (ILK4), potassium voltage-gated channel subfamily H member 5 (KCNH2) or ribonucleases P MRP subunit POP1 (POP1)(Table S5.3).

Table S5.3: List of significant SNPs linked with candidate genes

| Trait | SNPs | Position | Linkage group | Gene code | Gene name |
| --- | --- | --- | --- | --- | --- |
| Deformity |  |  |  |  |  |
|  | SNP26 | 56:C>T | NA | IK8 | Interleukin 8 isoform 1 |
|  | SNP30 | 66:T>C | NA | KCNH2 | Potassium voltage-gated channel subfamily H member 5 |
|  | SNP32 | 15:C>A | NA | POP1 | Ribonucleases P MRP subunit POP1 |

NA = Not available

The Interleukin-1 receptor-associated kinase 4 isoform X1 (ILK4) encoding a kinase that activates NF-kappaB in both the Toll-like receptor (TLR) and T-cell receptor (TCR) signaling pathways is essential for most innate immune responses. Another gene of interest is potassium voltage-gated channel subfamily H member 5 (KCNH2). Genome-wide association studies in human showed that this gene (KCNH2) is one of the candidates that could predispose to sudden cardiac death and drug-induced arrhythmias (Newton-Cheh et al., 2009). The encoded protein of the ribonucleases P MRP subunit POP1 (POP1) gene localizes to the nucleus and functions in pre-RNA processing and is also an autoantigen in individuals suffering from connective tissue diseases, such as lower jaw or nasal malformation in kingfish.

**References**

Newton-Cheh, C., Eijgelsheim, M., Rice, K.M., De Bakker, P.I., Yin, X., Estrada, K., et al. (2009). Common variants at ten loci influence QT interval duration in the QTGEN Study. *Nature genetics* 41(4)**,** 399-406.
