# Supplementary material for: First High-Density Linkage Map and Single Nucleotide Polymorphisms Significantly Associated With Traits of Economic Importance in Yellowtail Kingfish Seriola lalandi
Source: Front Genet. 2018 Apr 17;9:127. doi: 10.3389/fgene.2018.00127 (PMC5914296; doi:10.3389/fgene.2018.00127)
Supplement: FILE S1 — Sex-specific maker map for female and male yellowtail kingfish. [file Data_Sheet_1.zip › Data Sheet 1/Supplementary Files S1-7/Supplementary file S6. GWAS analysis for skin fluke.docx]

**Supplementary file S6. GWAS analysis results for skin fluke**

No significant SNPs were detected for skin fluke when high stringency *P*‑value less than < 5e^‑8^ (or -log10P >5) was used (Figure S6). However, when the significant threshold was set at 2×10^-4^ (or -log10P-value greater than 3), eight significant markers were detected to be associated with fluke disease from the mixed model analysis (Table S6). The number of significant markers obtained from GAT analysis were 8 and 4 from numeric regression with PCA corrections. Across the statistical models, only one SNP (CloneID: 13800544|F|0-46:G>A-46:G>A) was mapped to LG12. This marker explained about 4.3% of the total genetic variance in the model. There were about 27% of common markers detected from the three statistical methods (GAT, PCA-based regression and mixed model methodology).

| \|  \| \| --- \| \|  \| \| 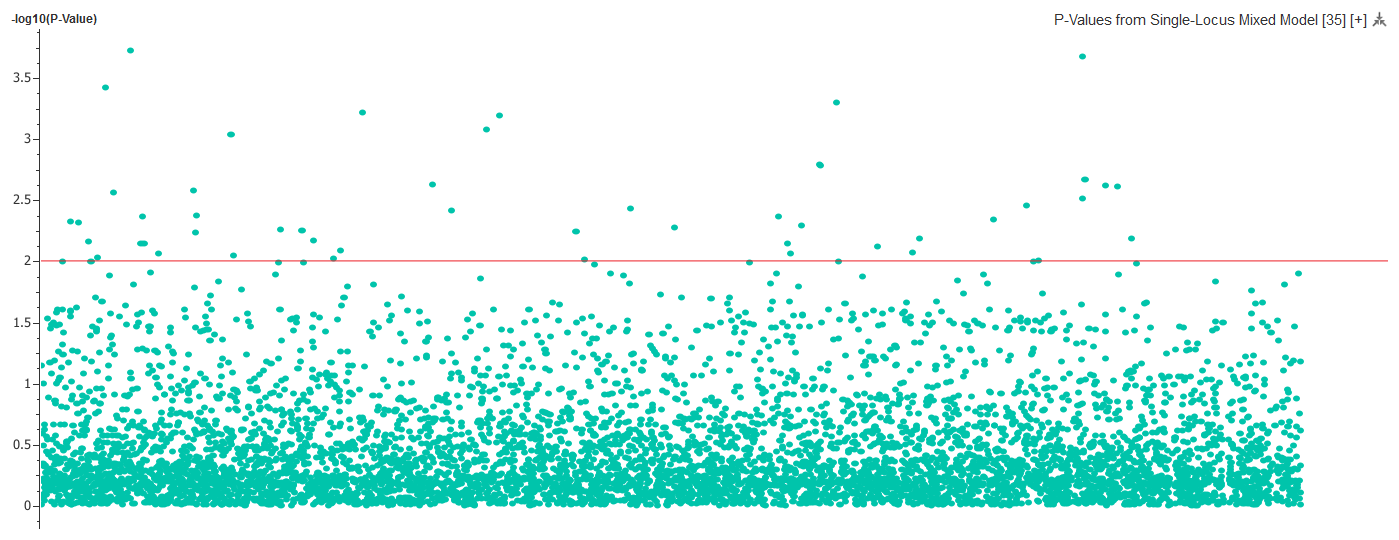 \| \| Figure S6: The Manhattan plot showing the –log10 (P-values) of SNPs on skin fluke using single locus mixed model \| |
| --- | --- | --- | --- | --- |
|  |

Table S6: Number of SNPs (N) and their false discovery rates FDR (%) for skin fluke (P < 5e^-4^ or

–log10P > 3)

| Trait | Correlation | | With PCA corrections | | Regression with PCA corrections | | Single locus mixed model | | Multiple locus mixed model | |
| --- | --- | --- | --- | --- | --- | --- | --- | --- | --- | --- |
|  | N | FDR | N | FDR | N | FDR | N | FDR | N | FDR |
|  |  |  |  |  |  |  |  |  |  |  |
| Skin Fluke | 27 | 1 | 27 | 1 | 22 | 0.92 | 8 | 0.99 | 8 | 0.99 |
