# Supplementary material for: First High-Density Linkage Map and Single Nucleotide Polymorphisms Significantly Associated With Traits of Economic Importance in Yellowtail Kingfish Seriola lalandi
Source: Front Genet. 2018 Apr 17;9:127. doi: 10.3389/fgene.2018.00127 (PMC5914296; doi:10.3389/fgene.2018.00127)
Supplement: FILE S1 — Sex-specific maker map for female and male yellowtail kingfish. [file Data_Sheet_1.zip › Data Sheet 1/Supplementary Files S1-7/Supplementary file S7. List of Significant SNPs Linked with Known Functions.docx]

**Supplementary file S7**: List of significant SNPs linked with candidate genes with known functions

| SNP | Gene symbol | Description |
| --- | --- | --- |
| **Weight** |  |  |
| SNP4 | CEP170 | centrosomal protein of 170 kda isoform x1 |
| SNP19 | HERC1 | probable e3 ubiquitin-protein ligase herc1 |
| SNP25 | LOC107376625 | voltage-dependent p q-type calcium channel subunit alpha-1a-like |
| SNP26 | PATL1 | protein pat1 homolog 1 isoform x1 |
| SNP32 | MR1 | major histocompatibility complex class i-related gene |
| SNP40 | STRA6 | stimulated by retinoic acid gene 6 protein homolog |
| SNP45 | LOC109136680 | dapper 1-like |
| SNP55 | LOC105022003 | lipase member h-like |
| SNP58 | LOC101156962 | serine threonine-protein kinase lmtk1- partial |
| SNP59 | MURC | muscle-related coiled-coil |
| SNP67 | LOC109643124 | leukotriene b4 receptor 1-like |
| SNP71 | LOC103471812 | heterogeneous nuclear ribonucleoprotein a0-like |
| SNP | TRIOBP | TRIO and F-actin-binding |
|  |  |  |
| **Deformity** |  |  |
| SNP1 | LOC107387636 | ankyrin-3-like isoform x26 |
| SNP3 | MPC1L | brain mitochondrial carrier protein 1-like |
| SNP5 | LOC106584280 | transcription factor ap-1-like isoform x1 |
| SNP6 | LOC101172156 | fibulin-7-like isoform x2 |
| SNP7 | PTPRM | receptor-type tyrosine-protein phosphatase mu isoform x2 |
| SNP18 | CEP170 | centrosomal protein of 170 kda isoform x3 |
| SNP26 | IK8 | interleukin 8 isoform 1 |
