# Supplementary figures and images for: First High-Density Linkage Map and Single Nucleotide Polymorphisms Significantly Associated With Traits of Economic Importance in Yellowtail Kingfish Seriola lalandi
Source: Front Genet. 2018 Apr 17;9:127. doi: 10.3389/fgene.2018.00127 (PMC5914296; doi:10.3389/fgene.2018.00127)

| 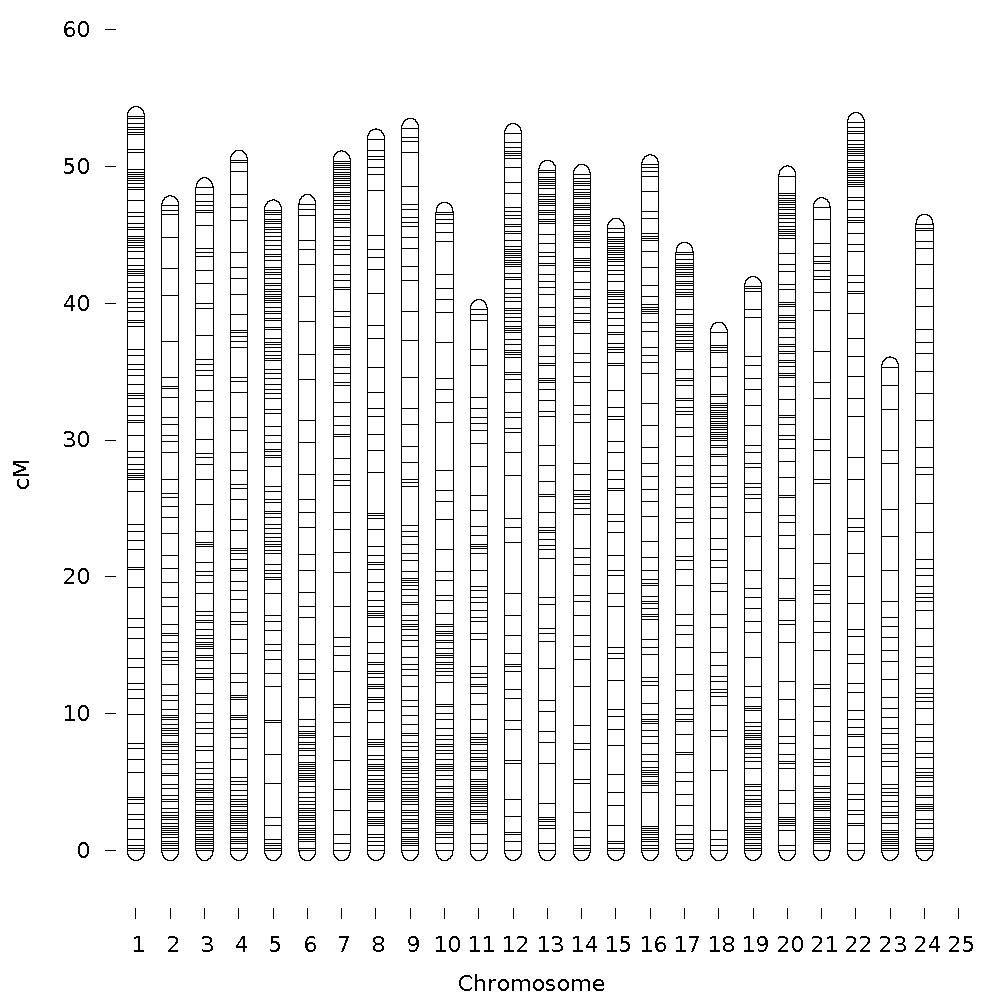 |
| --- |
| Male marker map |

| 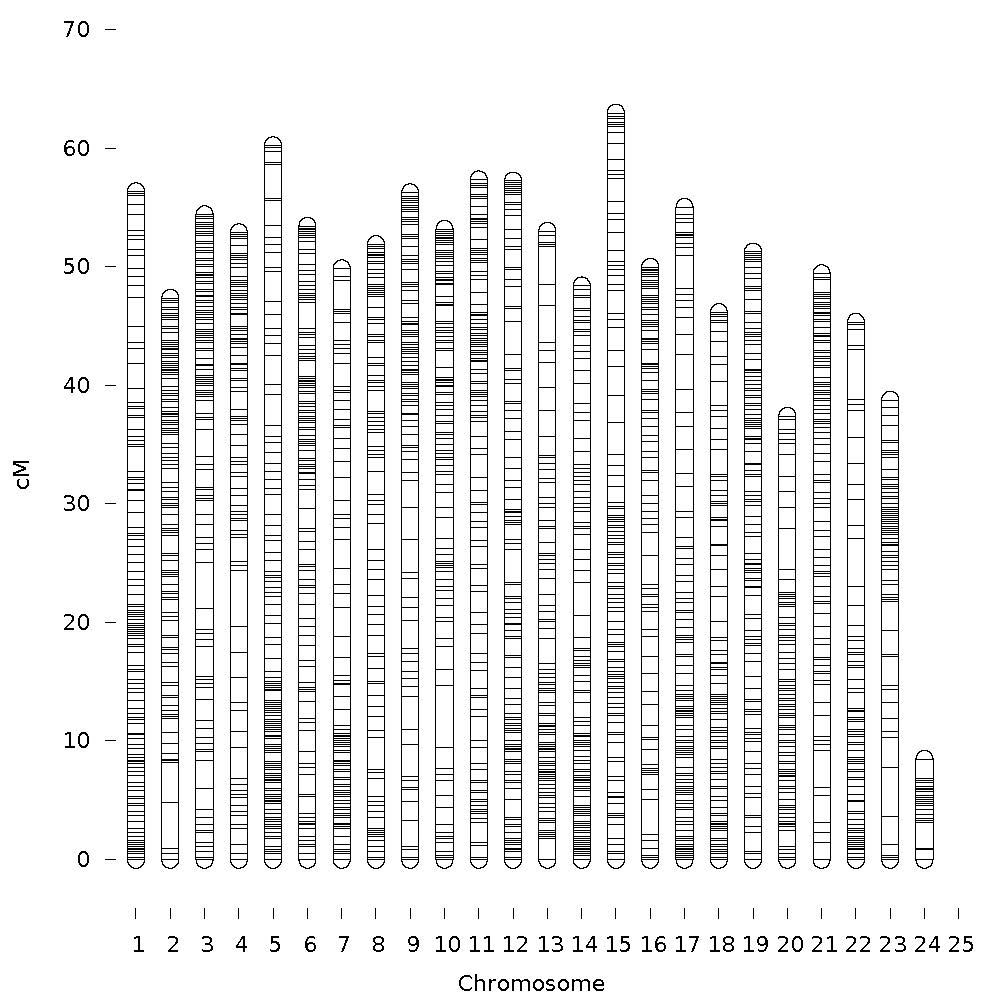 |
| --- |
| Female marker map |

Supplement: FILE S1 — Sex-specific maker map for female and male yellowtail kingfish. [file Data_Sheet_1.zip › Data Sheet 1/Supplementary Files S1-7/Supplementary file S1. Sex-specific maps.docx]
